# Anxiety and depressive disorders Screening among Healthcare Professionals

**DOI:** 10.1192/j.eurpsy.2023.1444

**Published:** 2023-07-19

**Authors:** W. Ayed, S. Chebbi, A. Ayadi, S. Ayari, H. Kebir, I. Magroun

**Affiliations:** Occupational health departement, University of Tunis El Manar - faculty of Medicine of Tunis, Ariana, Tunisia

## Abstract

**Introduction:**

Stress is an integral part of the profession of health care personnel (HCP) and manifests in higher rates of depressive and anxiety disorders (ADD).

**Objectives:**

Screening of anxiety and depressive disorders factors among HCPs

**Methods:**

A descriptive cross-sectional study in two university hospitals in Ariana was carried out ont september 2022. It included HCP who were examined for medical periodic visit. Data was collect from medical records, anxiety and depression Scale (HAD) and somnolence questionnaire (Epworth).

**Results:**

One hundred and nine HCP were included in the study. Women represented 87.2% of cases. The average age was 38 ±10.7 years. The average occupational seniority varied between one to 38 years. Nurses represented 38.5%, technicians 24% and doctors 7%. They had night work in 12% of cases. Depression and anxiety were found for 20% and 31% of cases respectively. Successive daytime sleepiness was found in 7% of cases. A statistically significant relationship was found between excessive daytime sleepiness and anxiety (p=0.005) and between depression (p=0.002).

**Conclusions:**

Anxiety and depressive disorders in HCP were considerable. they were assiciated with sleepiness disorder. Night or day time shift wasn’t statistically correlated with ADD. Referral to psychiatric consultations after psychological opinion was done in order garantee therapeutic support and decide fitness to work.

**Disclosure of Interest:**

None Declared

